# Advice for Food Systems Governance Actors to Decide Whether and How to Engage With the Agri-Food and Beverage Industry to Address Malnutrition Within the Context of Healthy and Sustainable Food Systems Comment on "Challenges to Establish Effective Public-Private Partnerships to Address Malnutrition in All Its Forms"

**DOI:** 10.34172/ijhpm.2021.70

**Published:** 2021-07-06

**Authors:** Vivica I. Kraak

**Affiliations:** Department of Human Nutrition, Foods, and Exercise, College of Agriculture and Life Sciences, Virginia Polytechnic Institute and State University, Blacksburg, VA, USA.

**Keywords:** Partnerships, Engagement, Malnutrition, Healthy Diets, Sustainable Food Systems, Planetary Health

## Abstract

The effectiveness of public-private partnerships (PPPs) to address malnutrition will depend on the issue, engagement purpose, policy context and actors’ interactions. This commentary offers advice for governments, United Nations (UN) and civil society organizations to decide whether and how to engage with industry actors to improve diets for populations. First, food systems governance actors must acknowledge and reconcile competing visions, harmonize numerous corporate-engagement principles, and support a shared narrative to motivate collective actions toward healthy sustainable diets. Second, food systems governance actors have tools to guide engagement through many alliances, networks, coalitions and multi-stakeholder platforms with different levels of risk and trust. Third, food systems governance actors must prioritize accountability by setting corporate-performance threshold scores to justify private-sector engagement; evaluating engagement processes, outcomes and consequences; using incentives, financial penalties and social media advocacy to accelerate time-bound changes; and revoking UN consultative status for corporate actors who undermine healthy people and planet.

## Introduction

**Figure 1:**
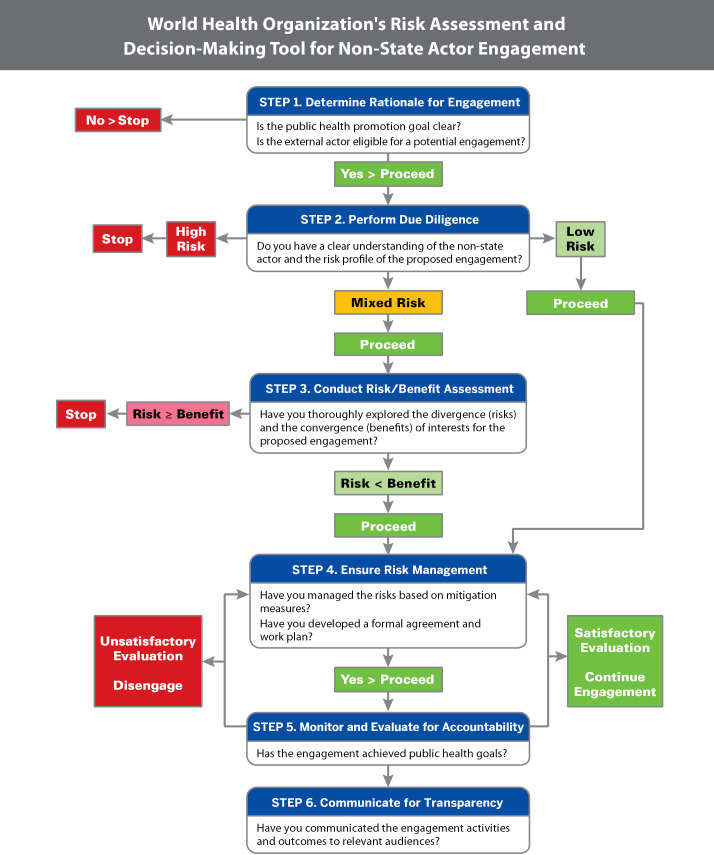
The WHO’s Risk-Assessment Tool for States to Guide Engagement with Non-State Actors to Address Malnutrition in All Forms. Adapted from WHO.^1^ Abbreviation: WHO, World Health Organization.

The effectiveness of voluntary public-private partnerships (PPPs) to address malnutrition will depend on the issue, purpose of engagement, policy context and use of risk-assessment and decision-making tools to guide actors’ interactions for the partnership process (Figure 1).^1^ There is currently no agreement among food systems actors—United Nations (UN) agencies, government and civil society organizations, and transnational agri-food and beverage firms—about how to achieve resilient food systems that provide safe, affordable, healthy, equitable and sustainable diets within the context of the Global Syndemic (ie, undernutrition, obesity and climate change) and coronavirus disease 2019 (COVID-19) pandemic. Many organizations use cross-sectoral partnerships legitimized and institutionalized over decades to achieve the UN Sustainable Development Goals (SDGs) Agenda.^2^ Business engagement may be pursued to address governance gaps, conduct research or implement programs to test bundled innovations, advocate for systems-level policy changes and improve corporate reputations.^2^

Fanzo et al^3^ conducted an evidence review as part of an independent evaluation funded by the Global Alliance for Improved Nutrition (GAIN), and analyzed 30 key informant interviews to understand the challenges associated with using PPPs to promote healthy diets within food systems to address malnutrition (ie, stunting, micronutrient deficiencies, obesity and diet-related non-communicable diseases [NCDs]) worldwide. They did not examine partnerships for agriculture and sustainable diets within the context of climate change. Their three major conclusions were that: (1) government and civil society organizations that engage with private-sector entities must address potential, perceived or actual conflicts of interest (COI); (2) substantial trust deficits exist between public- and private-sector actors that must be overcome; and (3) compelling evaluations are lacking to justify using partnerships over legislative or legal policy approaches to achieve diet, food system and health outcomes.^3^

This commentary offers advice for food systems governance actors to decide whether and how to engage with private-sector firms to address malnutrition within the context of healthy sustainable food systems. Strategies to identify, manage and mitigate nutrition-related COIs using the World Health Organization (WHO) risk-assessment tool^1^ were examined by Ralston et al^4^ and eight commentaries; therefore, COI is not addressed in this paper.

## Competing Visions and Paradigms for Healthy Sustainable Food Systems

Diverse stakeholders have competing visions, paradigms and narratives for establishing future sustainable food systems. By 2050, will local, regional, national and global food systems be healthy, green, equitable and sustainable for all people and the planet based on an optimistic, principled, inclusive and actionable vision?^5^ Or will people experience a market-driven dystopian future where food systems are governed by surveillance capitalism; and controlled by transnational technology, media, food and beverage, and chain restaurant firms with complicit governments that allow digital currency, precision agriculture, grocery deliveries via drones, and artificial intelligence-powered algorithmic nudging to collect and use people’s personal biometric information to influence what, when, where and how we eat?^6^

Myriad expert Lancet Commission reports and UN bodies, including the Food and Agriculture Organization (FAO) and WHO, have published guiding principles for state and non-state actors (ie, food and beverage firms, corporate and private foundations, and civil society) to support healthy sustainable diets.^7^ The UN’s Committee on World Food Security’s Voluntary Guidelines on Food Systems^8^ has developed a vision, roadmap and recommendations supported by 138 FAO member states^9^ that prioritize a human-rights approach, and identify partnerships as one of many potential forms of engagement with private-sector actors through multi-stakeholder deliberation platforms that will require transparency, democratic and strong accountable governance to produce desirable outcomes.^8^

Fanzo et al^3^ highlighted the need for prospective partners to differentiate between constructive versus rogue private-sector food and beverage industry actors, and identify effective policy approaches to reduce power asymmetries and hold firms accountable for practices that undermine healthy diets. Among the 30 informants interviewed were representatives of GAIN (the study funder) and the Scaling Up Nutrition (SUN) Business Network, co-convened by GAIN and the World Food Programme (WFP).^3^ But only two UN agencies—WHO and the United Nations Children’s Fund were interviewed and no representatives were included from the FAO, WFP, The World Bank or World Economic Forum to understand their position on partnerships used to address malnutrition.^3^

Controversy has simmered for decades about whether private funding and corporate philanthropy should be allowed to influence and erode the multilateral nature of UN agencies with dire implications for democracy within global governance.^2,10^ Civil society concerns have been raised about the 2021 UN Food Systems Summit that allege a “powerful alliance of corporations, philanthropies are subverting the multilateral UN institutions’ food governance to capture the global narrative of food systems transformation…and lack of transparency and accountability for how Summit participants are recruited and engaged to shape the agenda and outcomes to maintain corporate control of food systems.”^11^ One must acknowledge that that UN agencies had already deeply committed decades ago to use cross-sectoral partnerships to address many issues^2,10^ that impact malnutrition, diets and food systems.

The WFP supports PPPs to provide private-sector knowledge, resources and expertise to improve its impact, fundraising and innovation to achieve Zero Hunger by 2030.^12^ The WFP engages with Unilever and Yum Brands! (parent firm for KFC and Pizza Hut), without which this UN agency states it could not provide humanitarian support for vulnerable populations.^12^ The FAO also endorses a market-driven paradigm to justify partnerships to achieve the SDG 2030 agenda^13^ and has articulated engagement principles to justify corporate engagement for innovation, data sharing for dissemination, and support for financing and investment.^14^

GAIN, the SUN Movement’s Secretariat, and the SUN Global Business Network each have their own private-sector engagement principles to help build and sustain consumer demand for safe and healthy diets through several initiatives including the Making Markets Work program. The SUN Global Business Network’s 25 members have a combined revenue of over US$560 billion and Cargill, DSM, Kellogg, Mars, PepsiCo and Unilever provide technical assistance to countries through the network to design and implement food fortification, product development and reformulation, food safety and product marketing to populations in 63 low-income SUN countries. Four of these firms also participate in the International Food & Beverage Alliance (IFBA) that has adopted GAIN’s multi-stakeholder engagement principles, which do not mention protecting human rights from business impacts.^15^

 The market-driven paradigm embraced by the FAO, WFP, GAIN and the SUN Network reveal the inherent preference for a shared-governance approach as the most viable strategy to address hunger precipitated by humanitarian emergencies, undernutrition and micronutrient deficiencies for vulnerable populations especially in low-income countries. In response to COVID-19, many food and beverage firms donated funding to UN agencies, and products to charitable food providers to feed families who lost their income and livelihoods. These realities require critics of partnerships to identify viable alternatives and the trade-offs if UN agencies and national governments do not engage with businesses.

 A contrasting view describes transnational firms as having cultivated a neoliberal corporate paradigm that has favored corporate interests over public interests, and fostered policy inertia that has perpetuated a culture of overconsumption of unhealthy commodities and unsustainable systems that have driven the Global Syndemic. Yet the government regulation paradigm urged by civil society coalitions is inadequate to address the policy inertia.

 Systems change occurs at levels structural, relational and transformative. To achieve food systems transformation, governance actors must acknowledge and reconcile competing paradigms, harmonize corporate-engagement principles, and develop a shared vision and narrative with collective actions to motivate people to work together to promote health for all people and the planet given the major anthropogenic challenges that impede planetary health.

## Decision-Making Tools to Guide a Spectrum of Engagement With Industry Actors

Food system actors should use existing decision-making tools^1,4^ to clarify the purpose and guide a spectrum of engagement activities including networking, cooperation, coordination and collaboration that have different levels of risk and trust.The type of engagement requires understanding the issue and examining all available options and potential trade-offs, and preparing for anticipated and unintended outcomes, impacts and consequences. Research suggests that power-sharing and trust-building strategies built into engagement from the outset are important predictors of trust, and that power asymmetries and weak leadership are predictors of mistrust.^16^

**Figure 2:**
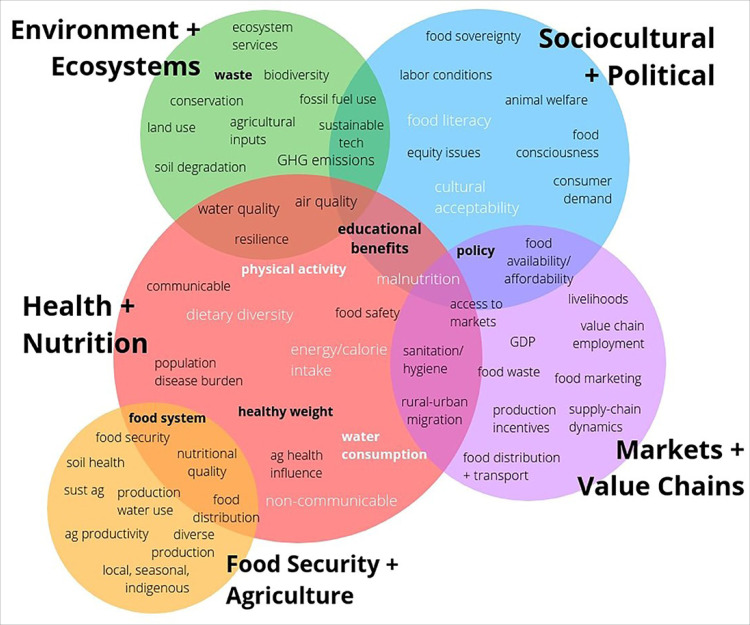
Five Dimensions of Sustainability Relevant to Healthy Diets and Food Systems Applicable to Private-Sector Engagement Commitments and Activities. Reprinted with permission from Mazac et al^17^ published by Frontiers. Abbreviations: GDP, gross domestic product; GHG, greenhouse gas.

**Figure 3:**
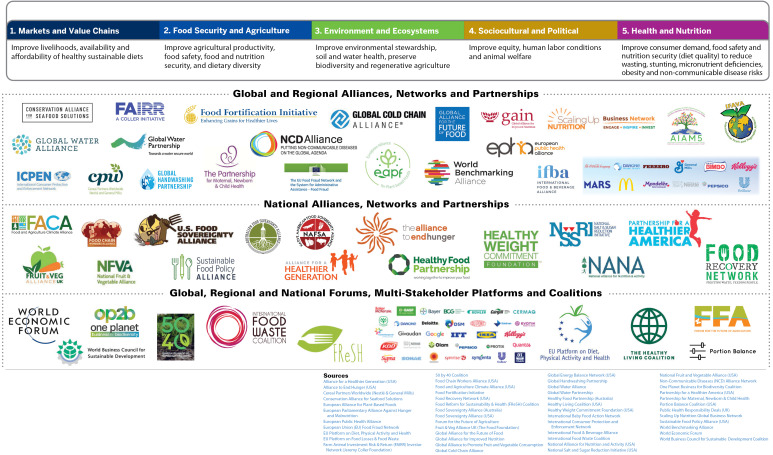
Examples of Actor Engagement Through Global, Regional and National Alliances, Networks, Partnerships, Forums, Platforms and Coalitions* that Promote Healthy and Sustainable Diets and Food Systems.
 *Published evaluations are limited for many of these alliance, partnership, network and coalition examples to assess whether their voluntary commitments have translated into policies and actions to support healthy and sustainable diets and food systems at local, national, regional or global levels. Further research and evaluations are needed to document outcomes achieved.
**Note**. The trademarked or copyright image use is allowed by the U.S. ‘nominative fair use’ legal doctrine that protects freedom of expression and speech by permitting the unlicensed use of copyright- or trademark-protected works in certain circumstances, such as when the intended use of the images is for non-commercial research and educational purposes only. For more information about Fair Use, please see the U.S. Copyright Office at https://www.copyright.gov/fair-use/more-info.html and U.S. Fair Use Evaluator at https://librarycopyright.net/resources/fairuse/index.php.

Fanzo et al^3^ have only scratched the surface of a complex topic that requires greater scrutiny to evaluate numerous global and national partnerships, alliances, networks, coalitions and multi-stakeholder platforms to facilitate food systems transformation within an institutional context.^1^ Real-world public- and private-sector engagement must be evaluated to assess whether these strategies are effective to achieve the SDGs in various country contexts. Figure 2 designed by Mazac et al^17^ illustrates five dimensions of sustainability applicable to private-sector commitments, engagement and activities that align with the UN Food Systems Summit’s five action tracks^[1]^. Figure 3 provides examples of more than 50 alliances, partnerships, networks, coalitions and multi-stakeholder platforms that address various dimensions of food systems sustainability but have different levels of risk and trust, and must be evaluated.

## Evaluation of Industry-Engagement Processes, Outcomes and Impact

Evaluations of partnerships in Australia, the United Kingdom and United States and the IFBA alliance of 12 global manufacturers^18^ suggest that many food, beverage and restaurant firms are setting less ambitious performance standards and time-bound targets recommended by expert bodies and UN agencies without consequences when they do not meet expected outcomes.

 Certain multi-stakeholder platforms and industry-funded networks have lost public trust and legitimacy, which are difficult to re-establish. Fifteen years after the European Union (EU) Platform on Diet, Physical Activity and Health was established to address obesity in 27 countries (2004-2019), the European Public Health Alliance and six civil society organizations jointly resigned, citing that the EU Platform was not fit for purpose and voluntary pledges were ineffective to reverse obesity and diet-related NCDs. The Multi-Stakeholder Initiative Integrity reached a similar conclusion in 2020 after evaluating 40 international standard bodies that were ineffective at restricting corporate power and holding businesses accountable for violating human rights.

 The Coca-Cola Company singularly funded the Global Energy Balance Network that advised physical activity researchers to minimize the contribution of sugary beverages to obesity and NCD risks. This industry front group was dissolved after investigative reporting and public outrage but has re-emerged. This example of corporate mis-behavior jeopardizes trust and legitimacy for future engagement, such as Coca-Cola’s support for Project Last Mile, a global partnership to strengthen health systems in three African countries.

These examples underscore the need for food system actors to build trusted relationships that will facilitate food system transformation, and ensure independent evaluations to document processes (ie, trust and synergy) and corporate-engagement outcomes and impacts. Governments must legislate and regulate industry practices to protect health, and also foster collaborative learning among actors to transform food systems. UN agencies and governments could more effectively hold industry accountable by setting corporate-performance thresholds to justify engagement such as an Access to Nutrition Index (ATNI) score of 8.0 or higher or requiring certified B Corporation membership^[2]^; using incentives and financial penalties to accelerate time-bound changes; and revoking UN consultative status for non-compliant industry actors who undermine human and planetary health. Civil society coalitions could use social media advocacy and activism to expose corporate public relations campaigns used to oppose government legislation to protect health; conduct external evaluations to ensure that corporate actors’ environmental sustainability pledges do not divert attention away from their human health commitments; expose greenwashing and bluewashing used to enhance corporate reputations; and use boycotts and shareholder advocacy to change corporate policies and practices.

## Conclusion

 To enhance the capacity of industry partnerships to promote healthy and sustainable diets and food systems to address malnutrition, there is a need for governance actors to clarify the policy context, purpose and rules of engagement, and assess the quality of actors’ interactions and outcomes. Food systems governance actors must acknowledge and reconcile competing visions and paradigms, harmonize numerous corporate-engagement principles, and develop a shared vision and narrative to motivate collective actions that create healthy sustainable diets within resilient food systems. Diverse actors should use existing decision-making tools to clarify goals and a spectrum of engagement activities with business actors, and use various accountability strategies including evaluations of engagement processes, outcomes and consequences that undermine healthy people and planet.

## Acknowledgments

I thank Juan Quirarte for creating Figures 1 and 3.

## Ethical issues

 Not applicable.

## Competing interests

 Author declares that she has no competing interests.

## Author’ contribution

 VIK is the single author of the paper.

## Disclosures

VIK participated in the study published by Fanzo et al.^3^

## Endnotes


[1] The UN Food Systems Summit 2021 has five action tracks (https://www.un.org/en/food-systems-summit/action-tracks) that include (1) ensure access to safe and nutritious food for all; (2) shift to sustainable consumption patterns; (3) boost nature-positive production; (4) advance equitable livelihoods; and (5) build resilience to vulnerabilities, shocks and stress.

[2] **The Access to Nutrition Index (ATNI) Initiative**https://accesstonutrition.org/ and **BIA-Obesity Tool **https://www.informas.org/bia-obesity/ rate food and beverage manufacturers firms on a scale of zero (no evidence) to 10 (best practice) based on their nutrition‐related commitments, performance, and disclosure across seven weighted categories including governance (12.5%), product formulation (25%), product accessibility (20%), responsible marketing (20%), healthy lifestyles (2.5%), food labeling (15%), and stakeholder engagement (5%). In 2018, the total ATNI score for the 12 IFBA firms ranged from 2.3 (General Mills) to 6.8 (Nestle) and no firm had achieved a score of 8 or higher out of 10.^18^ An updated ATNI report will be released in 2021. In 2020, ATNI Foundation released Investor Expectations for Nutrition, Diets and Health: https://accesstonutrition.org/investor-signatories/. **Certified B corporations** meet specific standards of verified social and environmental performance, public transparency, and legal accountability to balance profits and purpose. https://bcorporation.net/about-b-corps.

